# Detection of phylogenetically informative polymorphisms in the entire euchromatic portion of human Y chromosome from a Sardinian sample

**DOI:** 10.1186/s13104-015-1130-z

**Published:** 2015-04-30

**Authors:** Paolo Francalacci, Daria Sanna, Antonella Useli, Riccardo Berutti, Mario Barbato, Michael B Whalen, Andrea Angius, Carlo Sidore, Santos Alonso, Sergio Tofanelli, Francesco Cucca

**Affiliations:** Dipartimento di Scienze della Natura e del Territorio, Università di Sassari, Sassari, Italy; Center for Advanced Studies, Research and Development in Sardinia, Sassari, Italy; Institut für Humangenetik, Helmholtz Zentrum, Munich, Germany; Cardiff School of Biosciences, Cardiff University, Cardiff, UK; Istituto di Ricerca Genetica e Biomedica CNR, Cagliari, Italy; Departamento de Genética, Antropología Física y Fisiología Animal, Universidad del País Vasco, Bilbao, Spain; Dipartimento di Biologia, Università d Pisa, Pisa, Italy; Dipartimento di Scienze Biomediche, Università di Sassari, Sassari, Italy

**Keywords:** Human evolutionary genetics, Single nucleotide polymorphisms, Molecular phylogeny, Next Generation Sequencing

## Abstract

**Background:**

Next-Generation Sequencing methods have led to a great increase in phylogenetically useful markers within the male specific portion of the Y chromosome, but previous studies have limited themselves to the study of the X-degenerate regions.

**Methods:**

DNA was extracted from peripheral blood samples of adult males whose paternal grandfathers were born in Sardinia. The DNA samples were sequenced, genotyped and subsequently analysed for variant calling for approximately 23.1 Mbp of the Y chromosome. A phylogenetic tree was built using Network 4.6 software.

**Results:**

From low coverage whole genome sequencing of 1,194 Sardinian males, we extracted 20,155 phylogenetically informative single nucleotide polymorphisms from the whole euchromatic region, including the X-degenerate, X-transposed, and Ampliconic regions, along with variants in other unclassified chromosome intervals and in the readable sequences of the heterochromatic region.

**Conclusions:**

The non X-degenerate classes contain a significant portion of the phylogenetic variation of the whole chromosome and their inclusion in the analysis, almost doubling the number of informative polymorphisms, refining the known molecular phylogeny of the human Y chromosome.

**Electronic supplementary material:**

The online version of this article (doi:10.1186/s13104-015-1130-z) contains supplementary material, which is available to authorized users.

## Background

Knowledge of the evolution of the human genome depends on the availability of informative genetic markers to sustain phylogenetic reconstruction. In recent years, advanced genotyping technologies have enhanced the resolution of genome wide analyses by using hundreds of thousands (300 K to 650 K) of single nucleotide polymorphisms (SNPs) [1-3]. In recent years, data generated by large scale Next-Generation Sequencing (NGS) projects [4] have promised a fuller evaluation of genetic variation of the nuclear genome. However, for autosomes, genetic recombination, allelic gene conversion and natural selection complicate the phylogenetic reconstruction. Instead, because it does not recombine and has low reversion and recurrence rates, the male specific portion of the Y chromosome (MSY) can furnish key information about human evolutionary history.

The MSY consists of about 56.4 million base pairs (Mbp), excluding about 3 Mbp of the two telomeric pseudoautosomal regions (PAR) that recombine with the X chromosome. Only 23.1 Mbp have been mapped in the assembled human reference sequence (Hg 19, GRCh37), since the rest is made up of repetitive constitutive heterochromatin in the centromere and in the long arm of the chromosome that is essentially unreadable.

The majority of the euchromatic region falls into three classes [5]:

1. X-transposed sequences (3.4 Mbp), presenting a 99% homology to DNA sequences in Xq21, as a result of a X-to-Y transposition, occurring after the divergence of the human and chimpanzee lineages;
2. Ampliconic sequences (9.7 Mbp), with a marked self-identity prone to gene conversion and exhibiting, in the long arm, eight palindromes and two inverted repeats with 99.95% identity;
3. X-degenerate sequences (8.6 Mbp), with lower similarity with the X chromosome and encompassing single-copy gene or pseudogene homologues of different X-linked genes.

In addition, about 0.4 Mbp of euchromatic sequences could not be classified in any of the three classes and were labelled as “Other” [5].

Among the heterochromatic portion, about 1.0 Mbp of sequences, mainly located close to the centromere and in a small region interposed to two X-degenerate segments in the long arm, were sequenced in the GRCh 37 reference sequence release, raising the total amount of readable Y chromosome sequences to about 23.1 Mbp.

Some features of the X-transposed and Ampliconic classes (namely, marked homology and gene conversion) hamper their use for short-read sequencing, so published papers based on next generation resequencing have limited the study of MSY to the regions less prone to alignment problems. These studies applied similar and largely overlapping masks, encompassing chiefly X-degenerate sequences, which restricted the analysis to a range of about 9.1 Mbp [6,7] to 10.0 Mbp [8]. However, about 20% of the 1,749 markers of the human Y chromosome recognized by the International Society of Genetic Genealogy [9] at the end of 2012 (before the inclusion of SNPs derived from the aforementioned papers based on resequencing) falls outside the X-degenerate region, showing that other classes contain important phylogenetic information. In addition, some recent works in pre-print [10,11] report analyses of the whole readable stretch of MSY derived from publically available sequences of the 1000 Genomes Project. Thus, combined analysis of a larger set of informative markers in informative for populations could provide important insight into past demographic events. While it is easy to extract information with Sanger sequencing, as the procedure and sequence length enable unambiguous identification of the sequenced fragments, with next-generation short reads (100 bp), stringent mapping quality filtering is essential, together with a strict validation using phylogenetic criteria.

The present study aims to provide more coverage of MSY variation by extracting data from 23.1 Mbp of the Y chromosome in a representative sample of the isolated Sardinian population, already analysed with a filter of prevalently X-degenerate sequences [7], to improve the knowledge of the molecular phylogeny of the human Y chromosome.

**Figure 1:**
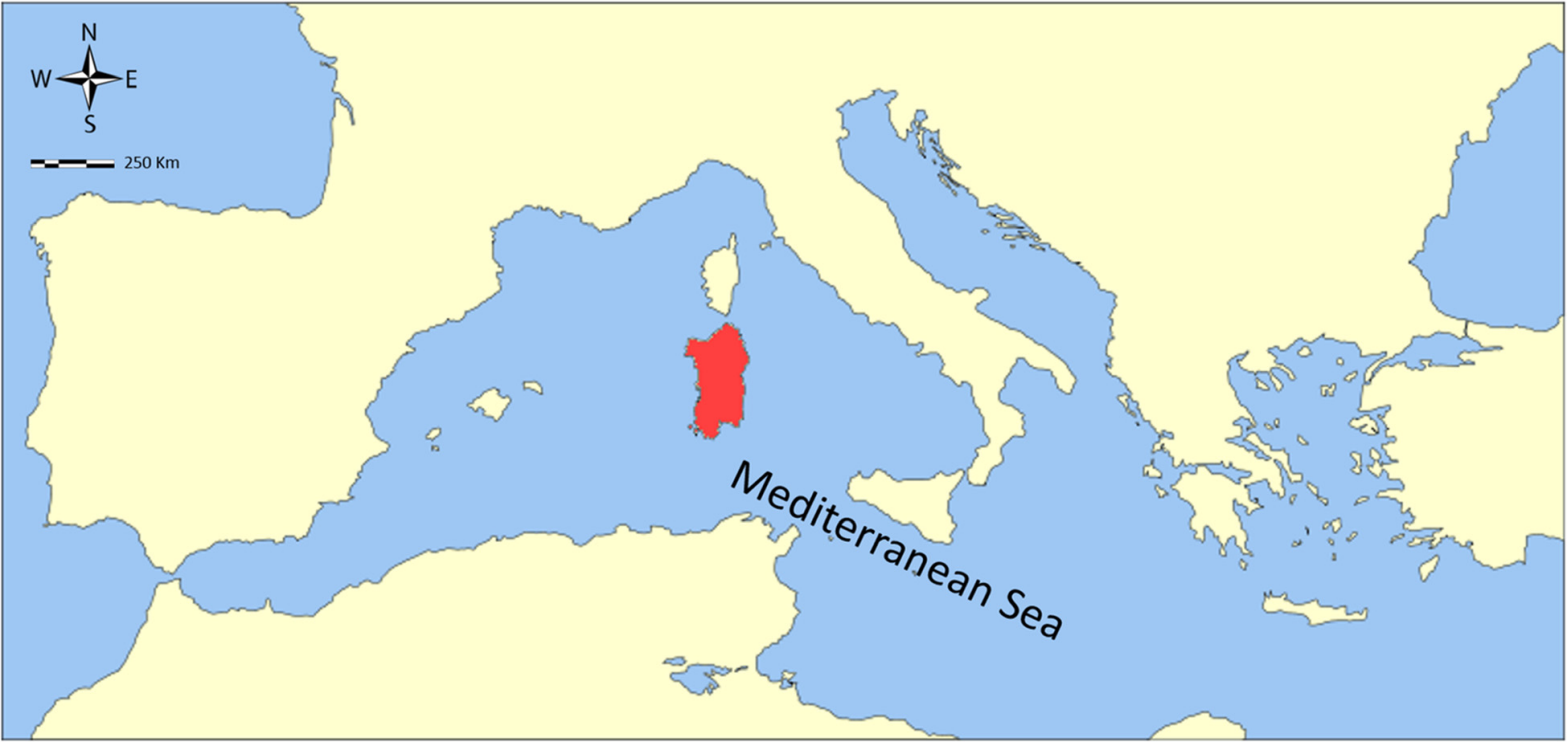
Geographic localization of the sample. The Mediterranean Sea with the island of Sardinia colored in red.

Sardinia, placed in the centre of Western Mediterranean sea (Figure 1), is of special interest for human geneticists, because it is a large genetic isolate with a high incidence of many heritable diseases and a peculiar distribution of alleles at multiple loci [12]. Some demographic and genetic features of this population, representing one of the main European genetic outliers together with Finland and the Basque Country, offered the opportunity to evaluate the potential impact of different evolutionary forces such as drift, inbreeding, gene flow and selection in an insular environmental context.

## Results and discussion

### Y chromosome polymorphisms

**Figure 2:**
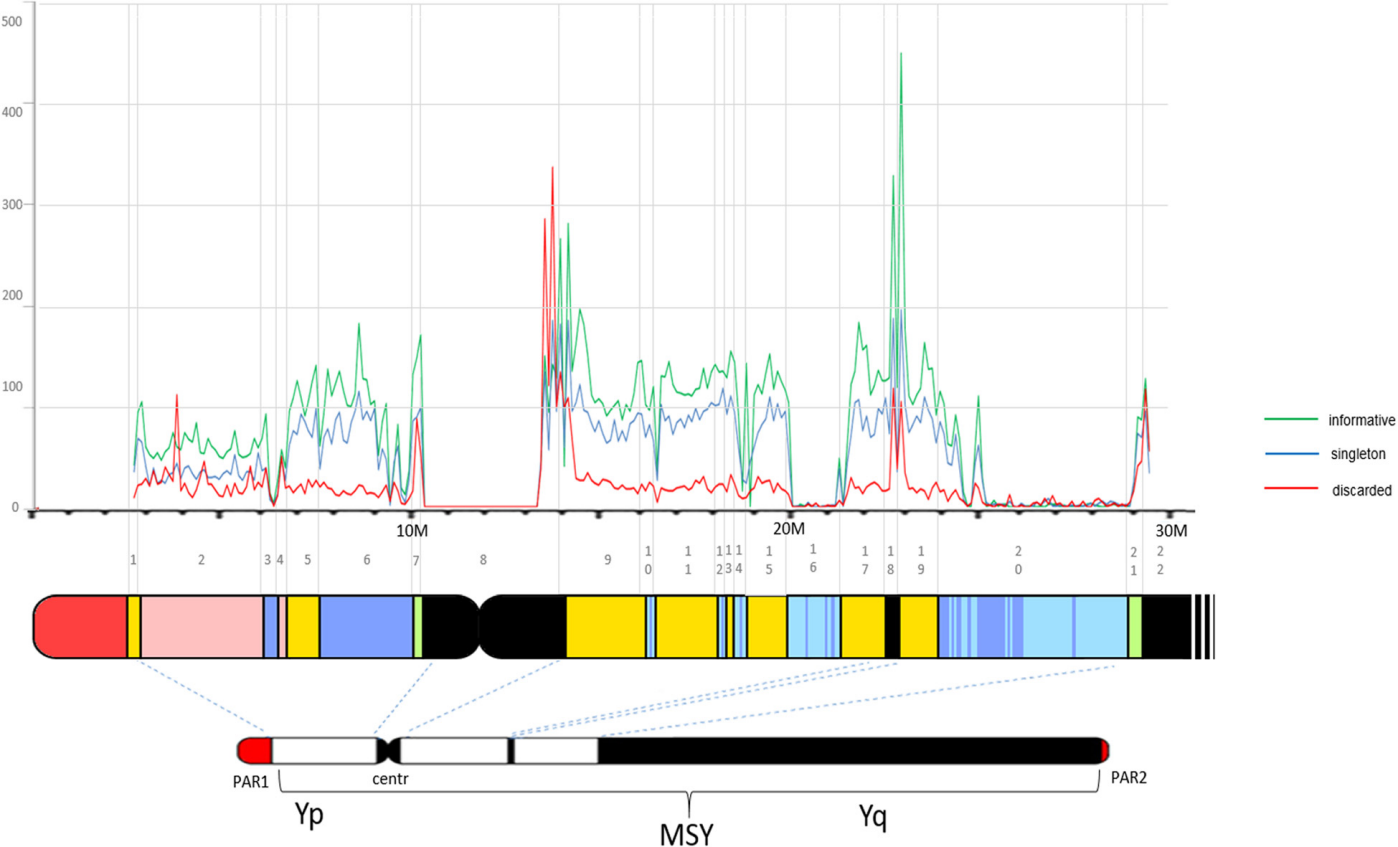
Physical map of Y chromosome sequence coverage and distribution of new informative SNPs. Schematic representation of Y chromosome including the pseudoautosomal (red) and heterochromatic regions (black) and enlarged view of the euchromatic portion of the MSY with different classes of euchromatic sequences: X-degenerate (yellow), X-transposed (pink), Ampliconic (blue), palindromes within Ampliconic classes (light blue), and Other (green). Redrawn from [7]. The lines indicates the distribution of Informative (green), Singleton (blue), and Discarded (red) SNPs along the molecule.

The analysis of 23.1 Mbp of the MSY of 1,194 Sardinian and 7 non-Sardinian samples yielded 39,277 polymorphic positions (Additional file 1: Table S1). Among them, 25,916 were present in at least two individuals or were already observed in other databases. After applying a hierarchical analysis, 20,155 (51.3%) were univocally associated with known haplogroups or sub-haplogroups, while 5,761 (14.7%) failed to show univocal association and were discarded for further analyses. The remaining 13,361 (34.0%) were singletons and were also excluded from the analyses. The filtered variants are unevenly distributed along the portion of the Y chromosome (GRCh37 assembly; Figure 2), extending from the proximal boundary of the Yp pseudoautosomal region to the proximal boundary of the large heterochromatic region of Yq, and were comprised of 54.4% in the X-degenerate class, 17.3% in the Ampliconic class, 11.2% in the X-transposed class, and 2.5% in none of these, marked as “Other”, while 14.5% were in the Heterochromatic region.

Although the X-degenerate class accounts only for 38.7% of the readable 23.1Mbp of the MSY, it contains 57.3% of the informative SNPs and 58.5% of the singletons, while the remaining 61.3% of the non-X-degenerate MSY classes accounts for 65.0% of the discarded SNPs (Table 1).

**Table 1:** Number of SNPs per Y chromosome regions

**Phylogenetic value**	**Class**	**n° SNPs**	**% SNPs**	**SNPs/10kbp**
	**X-degenerate**	**11547**	**57.3**	**13.49**
	**non X-degenerate**	**8608**	**42.7**	**5.90**
	● X-transposed	2216	11.0	6.52
Informative	● Ampliconic	3553	17.6	3.68
	*○ non palindromic*	*2613*	*13.0*	*9.27*
	*○ palindromic*	*940*	*4.7*	*1.38*
	● Other	442	2.2	11.26
	● Heterochromatic	2397	11.9	20.89
	**Total**	**20155**	**100.0**	**8.71**
	**X-degenerate**	**7815**	**58.5**	**9.13**
	**non X-degenerate**	**5546**	**41.5**	**3.80**
	● X-transposed	1209	9.1	3.56
Singletons	● Ampliconic	2474	18.5	2.56
	*○ non palindromic*	*1757*	*13.1*	*6.23*
	*○ palindromic*	*717*	*5.4*	*1.05*
	● Other	319	2.4	8.13
	● Heterochromatic	1544	11.6	13.44
	**Total**	**13361**	**100.0**	**5.77**
	**X-degenerate**	**2014**	**35.0**	**2.36**
	**non X-degenerate**	**3747**	**65.0**	**2.57**
	● X-transposed	981	17.0	2.88
Discarded	● Ampliconic	780	13.5	0.81
	*○ non palindromic*	*476*	*8.2*	*1.69*
	*○ palindromic*	*304*	*5.3*	*0.46*
	● Other	247	4.3	6.29
	● Heterochromatic	1739	30.2	15.16
	**Total**	**5761**	**100.0**	**2.49**

Number of SNPs, percentage of SNPs and number of SNPs/10 Kbp in the different classes of Y chromosome sequences subdivided for their relative phylogenetic value (Informative, Singleton, and Discarded). Bold numbers refer to the main Y chromosome regions (X-degenerate and non X-degenerate) and to the Total; regular numbers refer to the classes within the non X-degenerate region; italic numbers refer to the sub-regions within the Ampliconic class.

The NGS approach used, which relies on short reads, is not fully adequate to analyse region with marked homology, and a number of variants are expected to be lost during the aligning and filtering processes in these regions. This explains the observed heterogeneity in informative versus discarded variants along the chromosome. In particular, the Ampliconic class, which encompasses palindromic regions that hamper the unambiguous variant calling, yielded a very small number of informative variants.

When considering all the sequenced classes, the average number of derived alleles for each of the 1,194 individuals is 1,573.5 (±70.4) SNPs, with an increase of 574 SNPs with respect to the X-degenerate class alone.

Based on the low-pass complete genome sequencing from 1,194 individuals from Sardinia, we doubled the portion of the MSY analysed with respect to previous studies, significantly increasing the number of phylogenetically informative SNPs, and we constructed a more accurate phylogenetic tree of Y chromosome. Although the X-degenerate class of euchromatic sequences contains the majority of the phylogenetically informative signal, with an average of 13.5 informative SNPs/10Kbp (Table 1), we also show that also other classes can be effectively used to improve the evolutionary analyses. In particular, the heterochromatic portions proved to be very susceptible to variation, with almost 50 SNPs/10Kbp, and even if a significant portion of the polymorphisms were discarded for the lack of univocal association with known haplogroups, it still contains a remarkable density of phylogenetic information (20.9 SNPs/10Kbp). The aforementioned phylogenetic check is a necessary part of the filtering technique, since mapping quality alone is not sufficient to discriminate genuine Y chromosome variants from other sources of error. As a logical consequence, in the terminal branches of the tree, rarer variability will be partially lost. The Ampliconic class is the least informative, as expected because of its self-similarity and self-conversion hinder both the correct variant calling and the univocal association to haplogroups. However, the poor informative signal is mainly due to regions of the long arm of the chromosome, which contain several palindromes and inverted repeats, while those of the short arm show a similar intermediate behavior of other classes such as the X-transposed and the “Other”. In fact, the average density of informative SNPs in the palindromic regions is 1.4/10Kbp, whereas in the non-palindromic is 9.3/10Kbp (Table 1).

### Phylogenetic analysis on Sardinian samples

**Figure 3:**
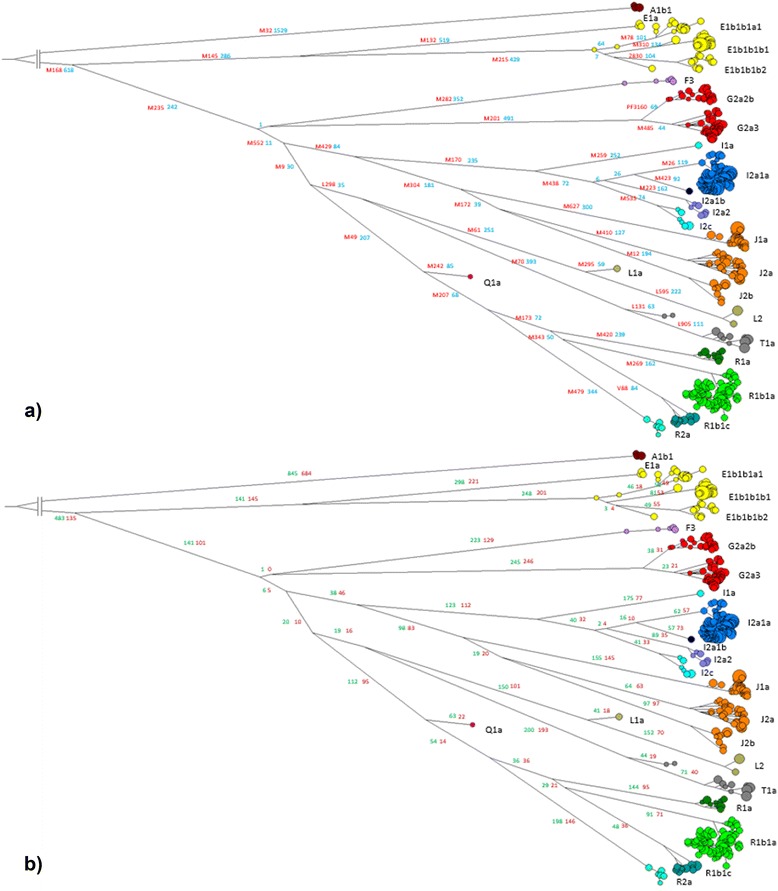
Phylogenetic network of the 1,194 samples. Median Joining network of the 1,194 Y chromosome sequences. The haplogroups are labelled according to the most recent ISOGG nomenclature. **a)** The main known marker is in red. The number of SNPs of each branch is in blue. **b)** The number of SNPs of each branch belonging to the X-degenerate class is in green. The number of SNPs of each branch belonging to the classes other than X-degenerate is in dark red.

The 20,155 validated SNPs were then used to construct parsimony-based phylogenetic trees. As shown in a schematic tree representation of the whole dataset (Figure 3a), all of the most common Y chromosome haplogroups (defined according to the ISOGG tree) that have been detected in Europe were present in our sample, with the sole exception of the northernmost Uralic derived haplogroup N.

To root the phylogenetic tree we used a *Pan troglodytes* sequence (see Methods) as outgroup, we placed the first bifurcation point within the dataset between individuals belonging to haplogroup A (samples 1–7) and the rest of the sample (samples 8–1,194), where the Most Recent Common Ancestor (MRCA) can be placed. It was not possible to infer the ancestral allele for 23 of 20,155 SNP positions, being the chimpanzee allele different from both reference and derived alleles detected in the human sample.

Currently, almost half of the discovered SNPs (8,862) make up the skeleton of the phylogenetic tree and constitute the root of the main clades. The skeleton comprises lineages that are unbranched for most of their length, with ramifications only in the terminal portion, according to an early separation of the clades and the sorting of ancient lineages followed by new variability generated during subsequent expansion events.

The addition of the phylogenetic information outside the X-degenerate portions is distributed rather proportionally along the branches of phylogenetic tree and its topology remains unaltered when the different classes of sequences are considered (Figure 3b), indicating the robustness of the phylogenetic inference.

**Figure 4:**
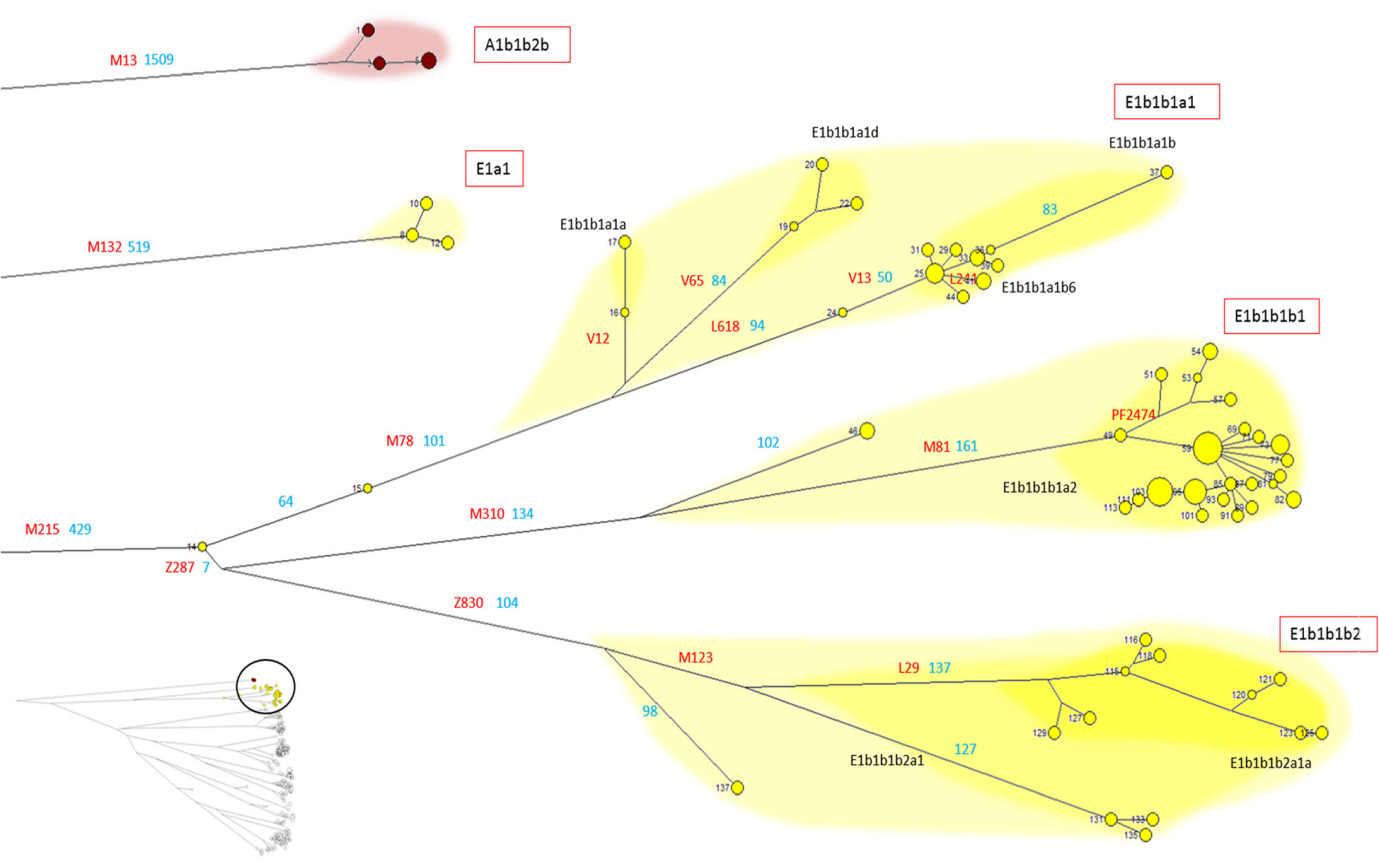
High resolution phylogenetic network of haplogroups A and E. The diameter of the circles is proportional to the number of individuals sharing the same sequence. The sample number is in black. In case of circles representing two or more samples only the first is reported. The branch length is proportional to the number of SNPs. In blue the number of SNPs for the main branches and for secondary branches longer than 50 SNPs. The known sub-haplogroups are labelled according to the most recent ISOGG nomenclature and represented with darker color. The labels of the main sub-haplogroups are framed in red. On the bottom left of the panel a small representation of the complete phylogenetic tree is reported. The circle indicate the position of the haplogroups in the general tree.

Seven individuals belonged to haplogroup A (samples 1–7) (Figure 4), a cluster of Y chromosome lineages common in the sub-Saharan area [13]. These Sardinian haplogroup A samples, like those detected in previous studies [12], were all characterized by the presence of the A1b-M13 mutation, with a predominantly East African distribution [14].

Overall, 131 individuals belonged to haplogroup E (samples 8–138) (Figure 4) distributed into four main clades. Six individuals (samples 8–13) belong to the sub-haplotype E1a-M33 (in its sub-haplogroup E1a1-M44), whose distribution is mainly Western African (Mali) [15]. The rest of the samples in this haplogroup belong to its European clade (E1b1b-M35). This haplogroup, common in Eastern Africa, is also widespread in the Mediterranean area [16].

**Figure 5:**
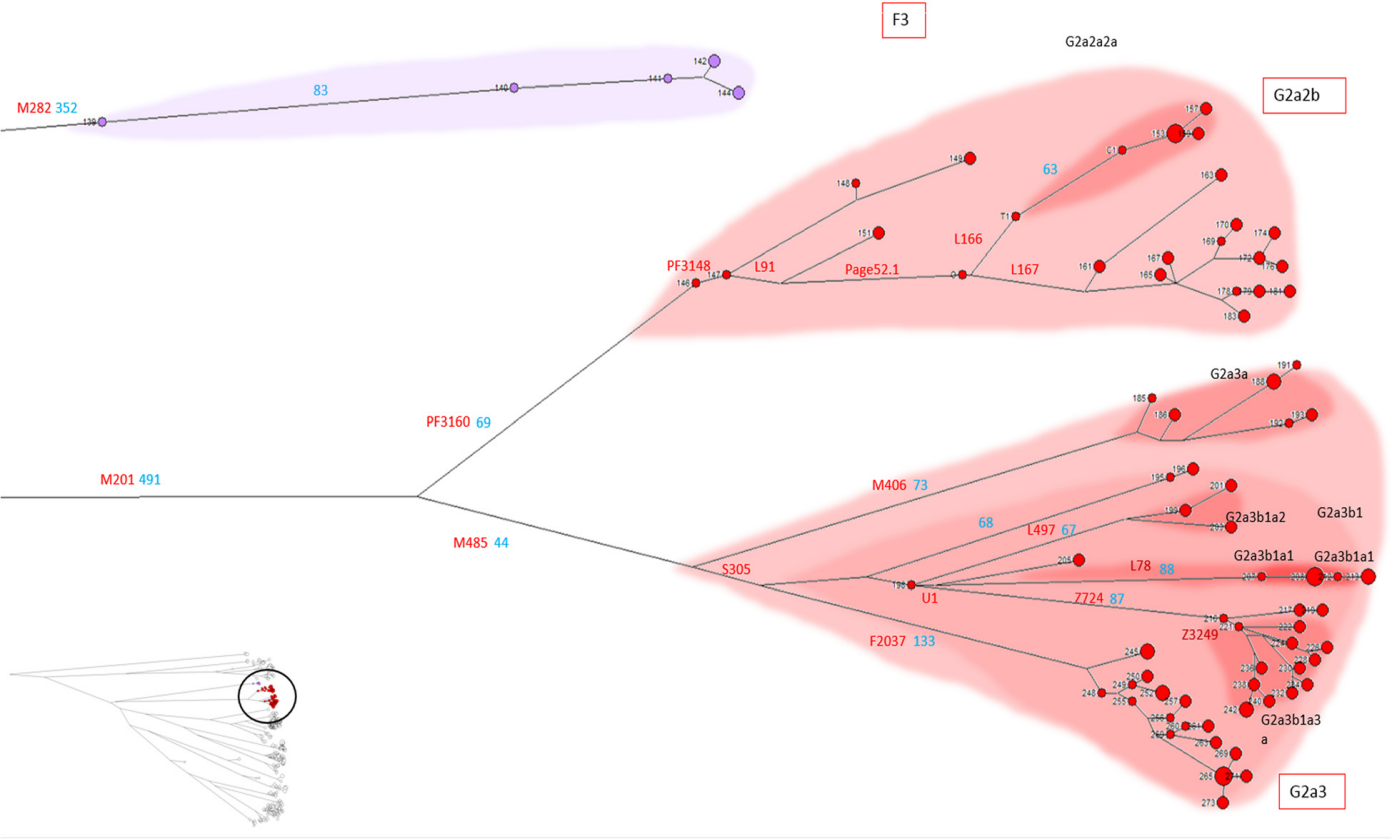
High resolution phylogenetic network of haplogroups F and G. Legend as in Figure 4.

The rare haplogroup F (samples 139–145) (Figure 5), which occurs sporadically in Europe [17], is present in 7 Sardinian samples in its sub-haplogroup F3-M481.

A total of 132 individuals belonged to haplogroup G (samples 146–274, including 1 Tuscan, 1 Corsican and the sequence of the Tyrolean iceman [18], the latter three out of the dataset numbering) (Figure 5). This haplogroup is otherwise restricted to the Caucasus, Near/Middle East and Southern Europe [19]. As previously reported [7], is also rather common in Sardinia.

**Figure 6:**
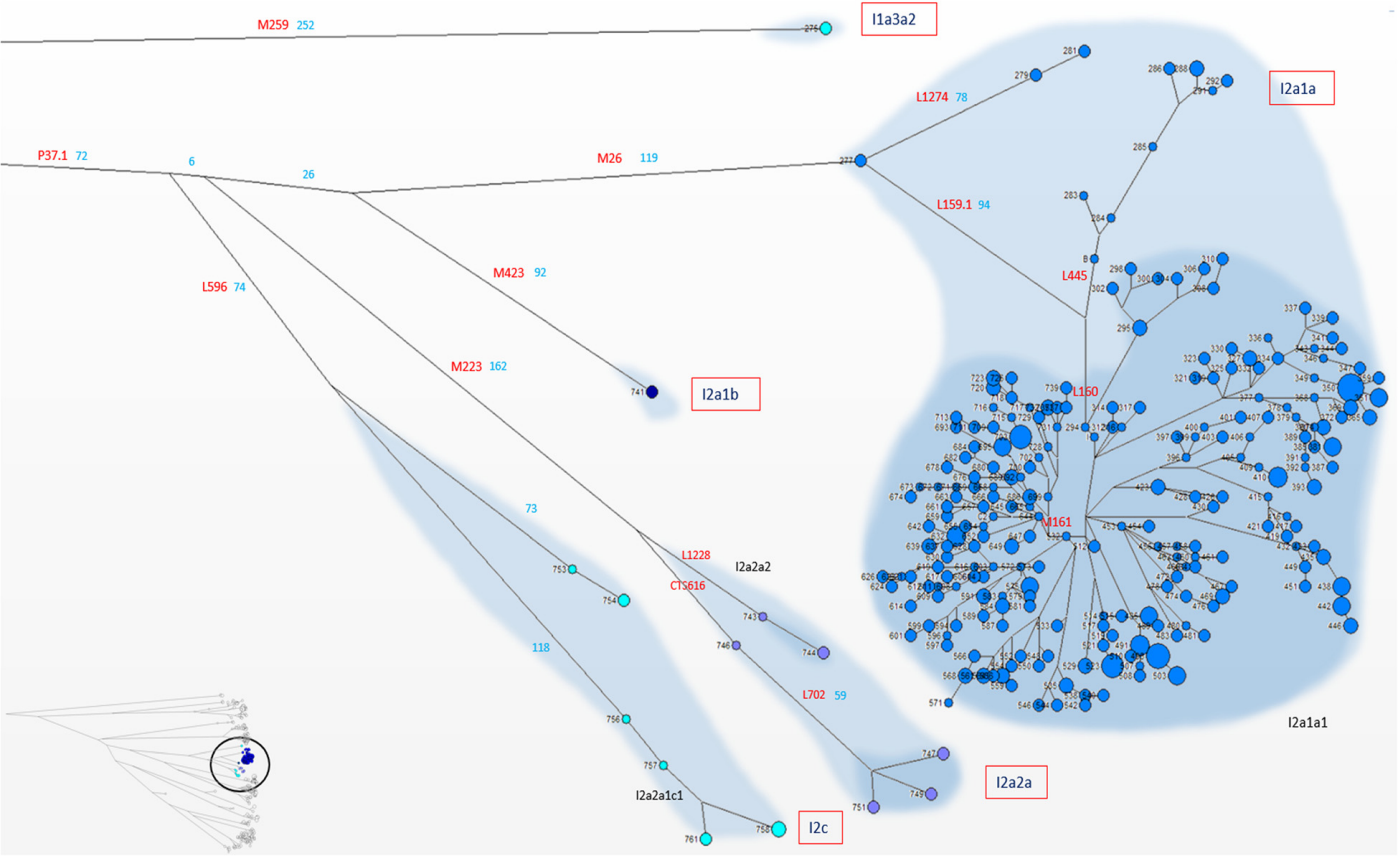
High resolution phylogenetic network of haplogroup I. Legend as in Figure 4.

Haplogroup I (samples 275–762) (Figure 6) comprises the majority of our sample, but relatively few individuals belong to I clades other than I2a1a-M26. In fact, I1-M253, associated with a Nordic diffusion given its high frequency in Fennoscandia [20], is represented by only two individuals (samples 275–276). Less rare are the sub-haplogroups I2c-L596 (samples 753–762), defined by L597, whose distribution indicates a possible origin in Central Europe, and I2a2a-M223 (samples 743–752) [21]. Sub-haplogroup I2a1-P37.2 is present in our samples in two clades, I2a1b-M423 (samples 741–742) and I2a1a-M26, reaching the percentage of 38.9% (samples 277–740, including 1 Basque, 1 Northern Italian and 1 Corsican). In agreement with previous observations, this latter sub-haplogroup is by far the most common in Sardinia [22,23]. Still, the distribution of I2a1a-M26 in Europe, and in particular the rare but constant presence in the Iberian Peninsula, with significant occurrence in Basques [24], suggests marking refuges during the last glaciation.

**Figure 7:**
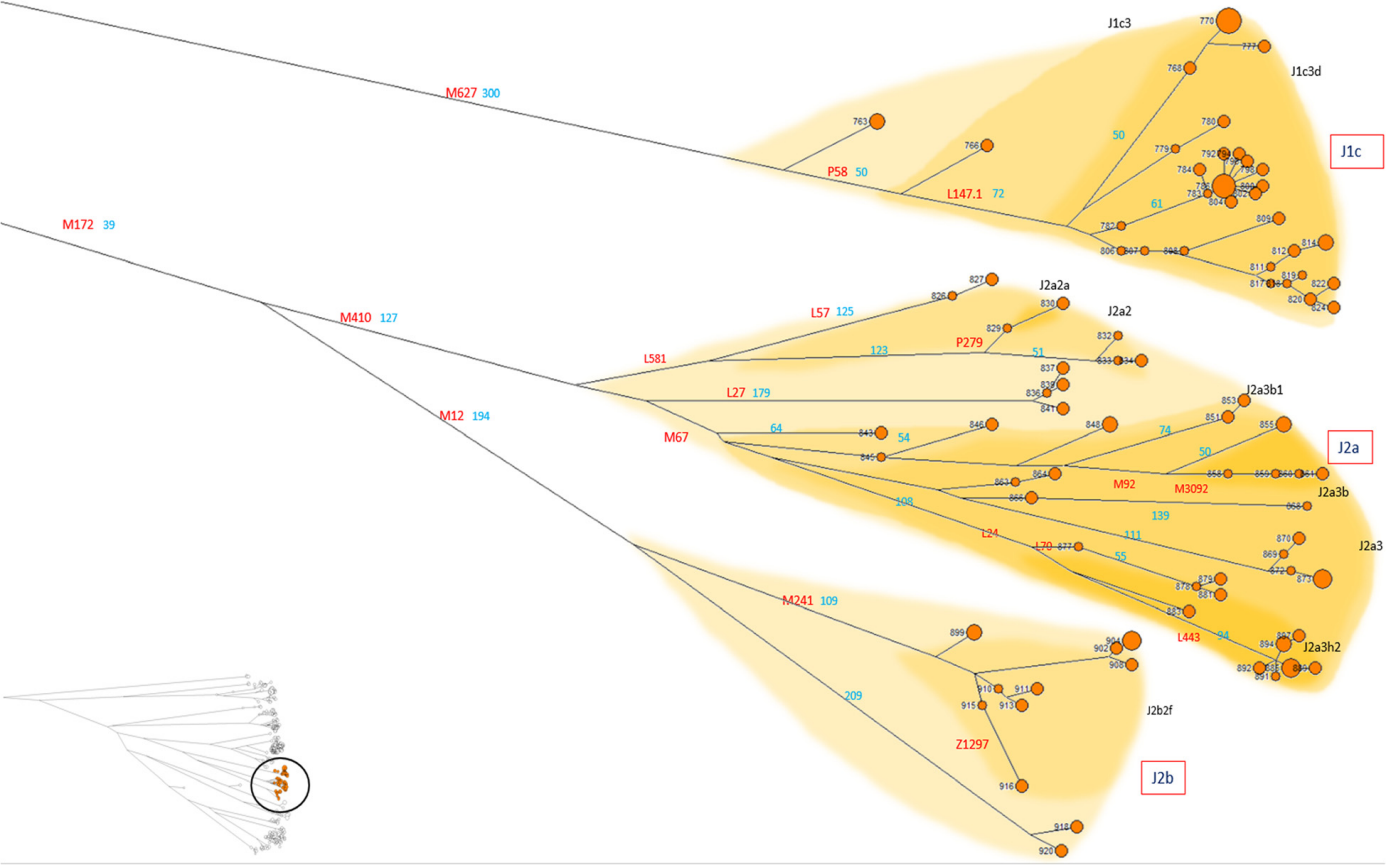
High resolution phylogenetic network of haplogroup J. Legend as in Figure 4.

Haplogroup J (samples 763–921) (Figure 7), a cluster of lineages with putative south-west Asian origin and diffusion [25] and with a significant presence in the Mediterranean area, was observed here with its main subgroups represented, J1c-M267 and J2-M172. The two sister clades, J1 and J2, have a dissimilar distribution, possibly reflecting different settlement pathways. J1-M267 has peaks in the Levant and in Northern Africa, while clade J2-M172 has higher frequencies in Anatolia and Mesopotamia, and decreases westwards [26].

**Figure 8:**
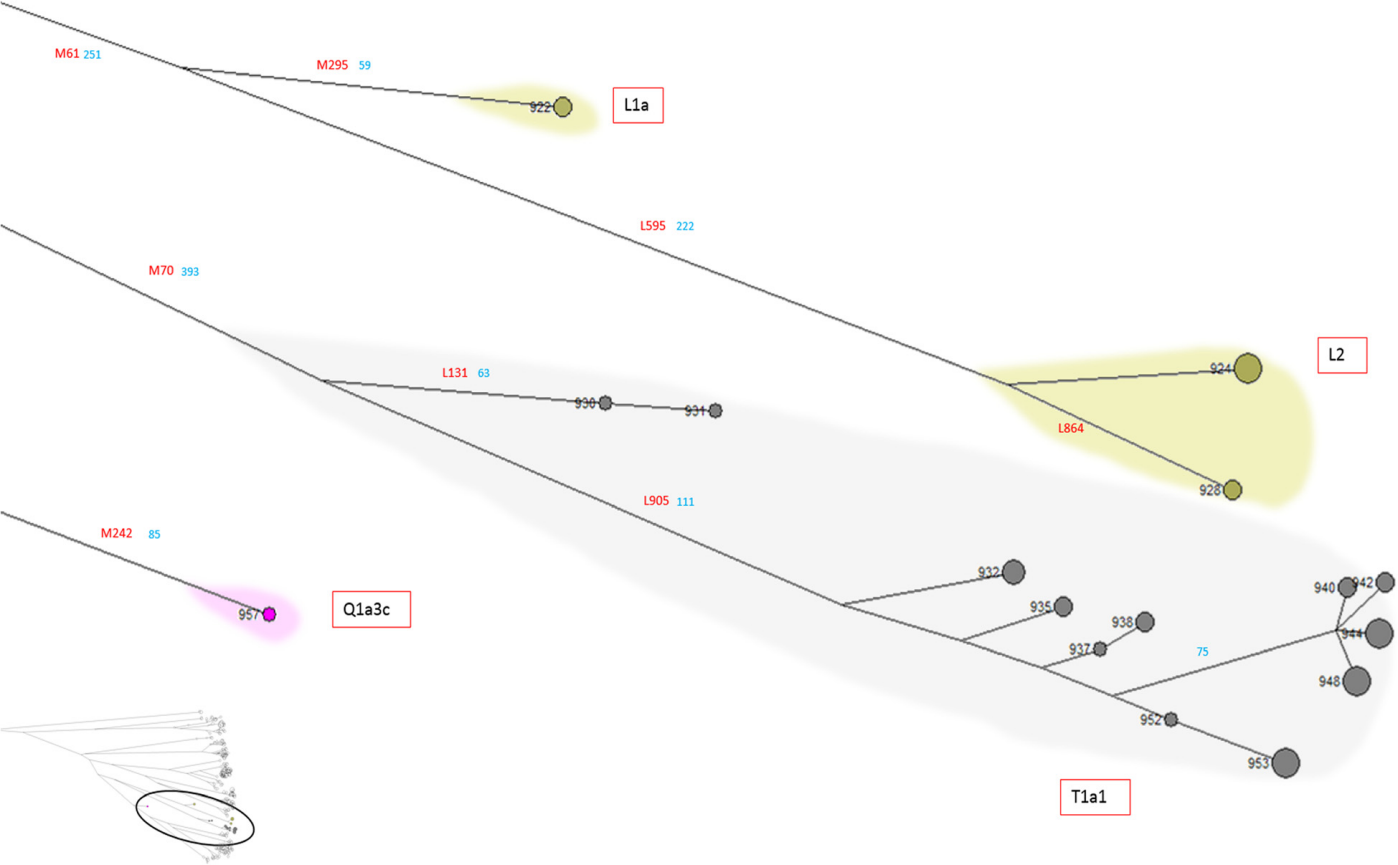
High resolution phylogenetic network of haplogroups L, T, and Q. Legend as in Figure 4.

The super-haplogroup K-M9, accounting for the rest of Sardinian samples, is present with P-L295 and LT-L298 branches; the latter represented by both L and T carriers. Haplogroup T (samples 930–956) (Figure 8), defined by mutation M70, is found at variable frequencies across West Asia, Africa, and Europe. Our sample shows two sub-haplogroups (T1a1-L905 and T1a2-L131). Only 8 individuals belong to haplogroup L (samples 922–929) (Figure 8), two of them in clade L1a-M27, scattered at low frequencies across Europe to the Indian subcontinent, where it reaches its highest frequencies [27], and the other in L2-L595, found only in Europe from Ireland to the Baltic [28].

The super-haplogroup P encompasses haplogroups Q and R, the former predominant among Amerindians, the latter representing the majority of European Y chromosomes. Haplogroup Q, present in our sample in a single individual (sample 957) and classified as Q1a3c-L527 (Figure 8), is rare in Europe, and according to Karafet et al. [17] might have originated in Central Asia.

**Figure 9:**
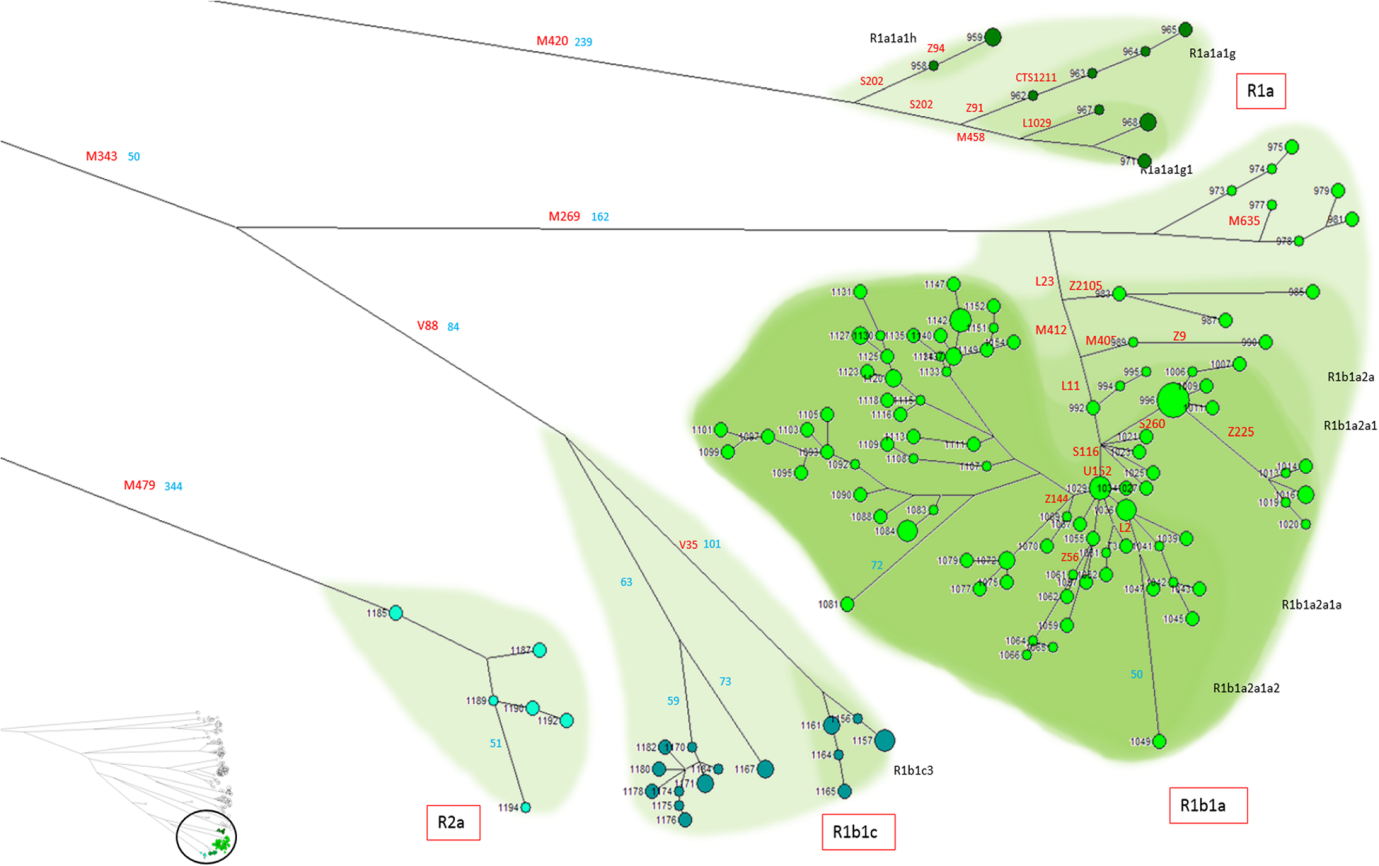
High resolution phylogenetic network of haplogroup R. Legend as in Figure 4.

Haplogroup R (samples 958–1194) (Figure 9) occurs mostly in its Western European branch R1b1a2- M269, but three other sub-haplogroups (R2a1-L295, R1a1a-M417 and R1b1c-V88) are also well represented. The sub-haplogroup R2a1-L295 (samples 1185–1194) is mainly present on the Indian subcontinent [27], and can be found in Europe in the Sinte Roma (Gypsies) of Indian origin [24]. The R1a1a-M417 subclade (samples 958–972) has its maximum occurrence in Eastern Europe, with frequencies over 50% among Slavic people. Its subclade R1a1a1b1a1 (formerly R1a1a7)-M458, present in our sample in 6 of 15 individuals (samples 967–972), has been linked to the spread of Bronze Age horsemen, associated with the Andronovo culture from the Central Asian steppe [29]. R1b1c-V88 (samples 1156–1184) has a mainly trans-Saharan distribution, except for the rare clade R1b1c1-M18 observed in Sardinia [30] and Corsica [31]. The 18 individuals classified R1b1c(xV35) (samples 1167–1184) very likely belong to the R1b1c1-M18 clade, although they cannot be positively identified in our dataset because the M18 marker is an In/Del polymorphism, not detectable with our analytical approach (see Methods). The most common haplogroup of Western Europe, R1b1a2-M269, encompasses 185 individuals (samples 973–1155, including 2 Tuscan samples). Its frequency in Europe is clinal, with higher percentages in Northwest Europe [22]. This large haplogroup is further subdivided into a number of subclades, many of them identified by SNPs detected in our sample. In particular, the sub-clade R1b1a2a1a1b2-U152, present in our sample in 129 individuals (samples 1029–1155), shows many private Sardinian lineages and has its peak frequency in Northern Italy/France [32].

The presence of various private Sardinian clades with star-like topology and different average branch lengths could be interpreted as reflecting the occurrence of some expansion phases of the Sardinian population. Notably, the clades with the higher average branch length (namely four clades of I2a1-M26 and one of G2a2b-L91) may represent the first population expansion that occurred on the island. In particular, the four I2a1 lineages, whose closer relatives can be found in Iberia (Basque Country), seems to be the descendant of the first Mesolithic settlers [19] that expanded following the acquisition of farming and pastoralism cultures [26]. The G2a2b-L91 lineage, which expands downstream to some non-Sardinian samples (Ötzi, a Tuscan and a Corsican, in this order) could represent the Neolithic newcomers to the island. In fact, the sequence of the naturally mummified sample (Ötzi) who lived in the Eastern Alps during the Copper age about 5,200 ya (years ago), has a coalescent age with the Sardinian G2a2b-L91 samples of about 9,000 ya [7], placing it among the common ancestors coming from the Caucasus and moving westward during the Neolithic [18].

Other clades with shorter average branch length, such as some sub-haplogroups of E (samples 115–130 = 51.3 SNPs and 49–114 = 24.9 SNPs), R (973–982 = 33.6 SNPs and 983–1155 = 39.9 SNPs) and G (161–184 = 46.9 SNPs and 245–274 = 53.0 SNPs), show a Sardinian private variability consistent with further expansion in the Late Neolithic (~5,500 to 6,000 BP), well documented by the Ozieri culture, and in the Bronze Age Nuragic period (~4,800 to 2,900 BP) [33].

Specific sub-haplogroups support the contact of Sardinia with both neighboring and distant populations. The presence in our sample of R1b-U152-L2 haplotypes, very common in central-northern Italy [32], may be interpreted as the long standing relationships of the island, starting from the Etruscan period to recent historic times, with populations from the coastal area of Tuscany and Liguria, while the R1a-M17/M458 haplotypes appear to be the westernmost descent of the people carrying the Indo-European languages [29]. The A3b2-M13 sub-haplogroup, found in 7 Sardinian individuals, shows an average length of private SNPs of 21.1 (±2.7). It has been reported in Sudan and it might have been imported into Sardinia by the Romans through the slave trade, analogously to what hypothesized elsewhere for the sporadic presence of another clade of haplogroup A (namely A3-M31) in England [34]. The other predominantly African sub-haplogroup E1a1-M44, frequent in West Africa and represented by 6 samples, shows an average branch length of 10 (±2.3) SNPs. This might be consistent with a founder effect related to the Vandals, who relocated a large number of males from the Mauritanian region into Sardinia as mercenary troops, as confirmed by historical sources. Other important haplogroups such as R2-M479, whose closest relatives are in the Sinti Roma population, and I2c-L596, with a scattered distribution in Europe, point out the complex demographic history of Sardinia, isolated but centrally located in the Mediterranean and thus subject to many cultural and genetic exchanges.

## Conclusions

In conclusion, extensive sequencing of the entire readable portion of MSY in this sample, followed by a hierarchical approach to detect biallelic markers, leads to significantly greater information about the molecular evolution of the human Y chromosome. The use of the complete 23.1 Mbp of the MSY, not restricted by mainly X-degenerate filters, almost doubles the number of available informative SNPs and increases the resolution of the phylogenetic tree, enhancing future comparative analyses. In fact, up to now the comparisons that can be carried out with most of the studied populations is only at the resolution level given by the detection of the common polymorphisms listed in ISOGG, which are located rather upstream in our phylogenetic tree. Moreover, we calculated mutation rate over this enlarged dataset, obtaining, as expected a higher value for the rate, accounting for an increased number of variants. It is also worth noting that variant density grew uniformly for all branches of the phylogenetic tree.

## Methods

DNA was extracted from peripheral blood samples of 1,194 adult males whose parents and grandparents were born in Sardinia. Because of the non-random nature of the sample (the sampling was primarily made with a biomedical aim), the data were used here for phylogenetic purposes, and no population analysis at sub-regional level was made. Seven other individuals from different European regions (1 Basque, 1 Continental Italian, 3 Tuscans, 2 Corsicans) of known haplogroup were added to the analysis. A published sequence of the so-called Iceman Ötzi [18] was also included.

The extracted DNA samples were prepared for sequencing, sequenced, genotyped and analysed for variant calling according to the methodology reported in [7]. The analytic approach applied to the sequencing data focuses on base pair substitutions (SNPs) and does not allow the detection of length polymorphisms such as STR and In/Dels. No position filter was applied, and all variants in respect to the GRCh37/hg19 reference sequence [35] comprised between positions 2,650,368-10,094,615 and 13,109,251- 28,818,849 were identified. The present analysis extends to approximately 23.1 Mbp the dataset reported in [7] and originally comprised of 1,204 Sardinian individuals. For 10 individuals, the available source data were restricted to the X-degenerate region, thus they were not included to avoid unwanted heterogeneity. Additional file 1: Table S1 (sheet 4) shows the conversion key of the sample numbering between the two datasets.

Sequence alignment was performed with bwa-0.5.9 [36], and variant calling with a modified version of glfMultiples [37] as described in [7].

A more strict mapping quality filtering (requiring 60 in the Phred scale, which means that both reads in a NGS read pair were aligned with no ambiguity) was applied to avoid carrying over reads from X chromosome.

The validated variants appearing in at least two individuals and univocally associate to known haplogroups, sub-haplogroup or phylogenetically related haplogroups [7], were considered informative. Variants present in single individuals were considered informative if already described in literature or in the ISOGG database as belonging to the same haplogroup of the individual sample. The polymorphic sites that were discovered in multiple individuals but could not be unequivocally assigned to any of the known haplogroups were discarded.

The lack of base call due to the absence of reads at a position in a particular sample was resolved either as an ancestral or derived allele by a hierarchical inferential approach as described elsewhere [7].

The ancestral status of each position was determined by comparison with a chimpanzee sequence using the LASTZ software as in the Ensembl-Compara pipeline [38] according to the method described elsewhere [6], integrated with data from Vaillant (SRX243490) [39]. The phylogenetically informative SNPs were used to build a phylogenetic unrooted tree using Fluxus-engineering Network 4.6 [40] according to the methodology described elsewhere [7].

### Consent

The present study was approved by the Institutional Review Board of the University of Cagliari. Each participant signed an informed consent form. In the case of newborns, consent was obtained from the child’s parents.

### Availability of supporting data

The data set supporting the results of this article is available in the European Genome-phenome Archive (EGA, www.ebi.ac.uk/ega/), which is hosted by the European Bioinformatics Institute, under accession number EGAS00001000532.

## Additional file

Additional file 1: Table S1.
**Sheet 1** – Informative (univocal) SNP list. Column A: SNP-ID. Column B: Y-chromosome region. Column C: Y-chromosome class. Column D: Physical position in GRCh37. Column E: Reference allele. Column F: Alternative allele. Column G: Ancestral allele. Column H: Haplotype assignment. Column I: First sample with the derived allele. Column J: Last sample with the derived allele. Column K: Non Sardinian samples with the derived allele (O = Ötzi T = Tuscan B = Basque C = Corsican I = Northern Italian). Column L: Total number of individuals with derived alleles. Column M: Percentage observed/total derived alleles. Column N: ISOGG marker code. Column O-Q: Alternative ISOGG marker code. **Sheet 2** – Singleton (private) SNP list. Column A: SNP-ID. Column B: Y-chromosome region. Column C: Y-chromosome class. Column D: Physical position in GRCh37. Column E: Reference allele. Column F: Alternative allele. Column G: Ancestral allele. Column H: Haplotype assignation. Column I: Individual #. Column J: The asterisk (*) indicates the position with 4 or more reads. Column K: ISOGG marker code. Column L: Alternative ISOGG marker code. **Sheet 3** – Discarded (non-univocal) SNP list. Column A: SNP-ID. Column B: Y-chromosome region. Column C: Y-chromosome class. Column D: Physical position in GRCh37. Column E: Reference allele. Column F: Alternative allele. Column G: total number of samples with the derived allele. Column H: ISOGG marker code. Column I: Alternative ISOGG marker code. **Sheet 4** - Conversion key of the individual numbers between the dataset reported in [7] and the present work. Column A: Individual # in [7]. Column B: Individual # in the present work. **Sheet 5** – Coordinates of the Y chromosome regions. Column A: physical position in GRCh37. Column B: number of the region. Column C: Y-chromosome class.

## Abbreviations

GRCh37: Genome Reference Consortium Human genome build 37
ISOGG: International Society of Genetic Genealogy
MRCA: Most recent common ancestor
MSY: Male specific portion of the Y chromosome
NGS: Next-Generation Sequencing
SNP: Single nucleotide polymorphism
STR: Short tandem repeats
